# Association of circulating PCSK9 concentration with cardiovascular metabolic markers and outcomes in stable coronary artery disease patients with or without diabetes: a prospective, observational cohort study

**DOI:** 10.1186/s12933-020-01142-0

**Published:** 2020-10-06

**Authors:** Jia Peng, Ming-Ming Liu, Jing-Lu Jin, Ye-Xuan Cao, Yuan-Lin Guo, Na-Qiong Wu, Cheng-Gang Zhu, Qian Dong, Jing Sun, Rui-Xia Xu, Jian-Jun Li

**Affiliations:** State Key Laboratory of Cardiovascular Diseases, Fu Wai Hospital, National Center for Cardiovascular Diseases, Chinese Academy of Medical Sciences and Peking Union Medical College, No 167 BeiLiShi Road, XiCheng District, Beijing, 100037 China

**Keywords:** PCSK9, DM, CAD, Maces, Cardiovascular metabolic markers

## Abstract

**Background:**

Whether plasma proprotein convertase subtilisin/kexin type 9 (PCSK9) levels is a predictor for cardiovascular outcomes has currently been controversial. No data is currently available regarding the relation of PCSK9 to cardiovascular metabolic markers (CVMMs) and major adverse cardiovascular events (MACEs) in stable coronary artery disease (CAD) patients with diabetes or without diabetes.

**Methods:**

A total 1225 untreated patients with stable CAD were consecutively enrolled and their baseline plasma PCSK9 levels were determined by ELISA. Patients were divided into high and low PCSK9 groups according to PCSK9 median. All patients followed up for the occurrence of MACEs and received standard therapy after admission. The associations of PCSK9 with CVMMs and MACEs were evaluated.

**Results:**

PCSK9 levels were positively correlated with multiple CVMMs including total cholesterol, low-density lipoprotein cholesterol, non-high-density lipoprotein cholesterol and hemoglobin A_1c_ at baseline (all *p* < 0.05). During a median follow-up of 3.3 years, 103 (8.4%) events occurred. PCSK9 levels were higher in patients with events compared to those without (*p* < 0.05). The Kaplan–Meier analysis displayed that patients in high PCSK9 group had lower event-free survival than that in low group (*p* < 0.05). Multivariable Cox regression analysis revealed that PCSK9 levels were independently associated with MACEs in diabetic patients (adjusted hazard ratio [HR]: 1.361, 95% confidence interval [CI]: 1.037–1.785, *p* < 0.05). When added the combination of PCSK9 levels and diabetic status to stratifying factors, patients in high PCSK9 group appeared to have extremely high risk of subsequent MACEs with diabetes (adjusted HR: 5.233, 95% CI: 2.546–10.757, *p* < 0.01).

**Conclusions:**

The present study firstly showed that elevated PCSK9 levels were related to multiple CVMMs and MACEs in stable CAD with diabetes, suggesting that plasma PCSK9 measurement could help to identify diabetic patients with CAD at higher cardiovascular risk. More studies may be needed to confirm our findings.

## Background

Diabetes mellites (DM) is regarded as coronary artery disease (CAD) equivalent and associated with an obvious increase in the risk of atherosclerotic cardiovascular disease (ASCVD) [1, 2]. Meanwhile, ASCVD-related mortality has been reported to be significantly higher in diabetic patients than that in nondiabetic patients (20 *vs* 3.5%) [3]. Additionally, DM is commonly accompanied with multifaceted metabolic disorders and the risk of ASCVD in patients with DM would be amplified by several concurrent atherogenic metabolic factors [4]. More importantly, atherogenic dyslipidemia is one of the major common clinical manifestations, which is an important predictive factor of cardiovascular risks in diabetic individuals. The management of this type of dyslipidemia, especially targeting low-density lipoprotein cholesterol (LDL-C) levels with optimal strategies, can significantly bring cardiovascular benefits in diabetic patients [5–7].

Proprotein convertase subtilisin/kexin type 9 (PCSK9), a serine protease belonging to the proteinase K subfamily of subtilases, is a critical player in cholesterol metabolism by increasing the degradation of hepatic low-density lipoprotein cholesterol receptors (LDLR), thereby resulting in increasing circulating LDL-C levels [8, 9]. Recently, a large number of studies have demonstrated that PCSK9 can directly or indirectly contribute to atherosclerosis from initiation to progression by leading to endothelial dysfunction, promoting inflammatory response and inhibiting platelet activation, which is independent on its effects on regulation of cholesterol metabolism [10–12]. Consequently, several large-scale event reduction trials have found that human PCSK9 monoclonal antibodies markedly reduce LDL-C levels and show a great promise for a reduction of future cardiovascular events [13, 14]. It is worth noting that circulating PCSK9 concentration has been proposed as an adverse factor for cardiovascular risks in patients with CAD [15, 16]. What’s more, a growing number of studies have explored the association of PCSK9 with cardiovascular metabolic disorders beyond dyslipidemia [17–19]. Nevertheless, the study on the association between plasma PCSK9 levels and cardiovascular metabolic markers (CVMMs) is limited in diabetic patients with CAD and whether plasma PCSK9 concentration can predict major adverse cardiovascular events (MACEs) in these patients has not been evaluated.

Hence, in the current study, we try to investigate the association of plasma PCSK9 levels at baseline with CVMMs and the future MACEs in stable CAD patients with DM or without DM, who did not take any lipid-lowering therapy before admission and received a standard treatment after admission.

## Method

### Study population

The present study protocol was complied with Declaration of Helsinki and was approved by the hospital ethics review board (Fu Wai Hospital & National Center for Cardiovascular Diseases, Beijing, China, approval number: 2013-442). Every patient signed informed written consent before enrolled in this study.

**Fig. 1:**
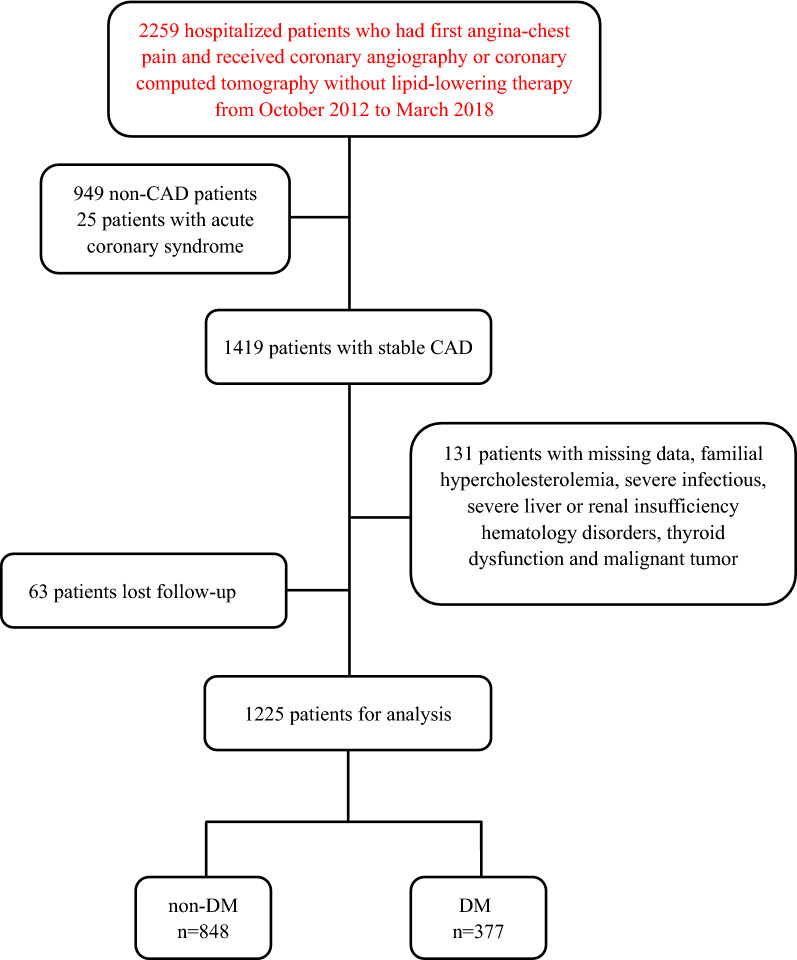
Flowchart of the study. *CAD* coronary artery disease, *non-DM* non-diabetes mellitus, *DM* diabetes mellitus

As shown in the flowchart (Fig. 1), from October 2012 to March 2018, 2259 patients with first angina-like chest pain, who took no lipid-lowering drugs within 3 months before admission were enrolled in the current study. All of them completed coronary angiography and/or coronary computed tomography to confirm their diagnosis of CAD. Among them, 949 patients without CAD (coronary stenosis ≥ 50% of at least one coronary artery) were excluded. Additionally, patients diagnosed as acute coronary syndrome or previous myocardial infarction (MI), patients without PCSK9 or detailed laboratory data, patients with familial hypercholesterolemia (FH), severe infection, severe liver or renal insufficiency (plasma creatinine < 150 μmol/L at baseline), hematology disorders, thyroid dysfunction and malignant tumor were also excluded from this study. Finally, 1288 patients firstly diagnosed as stable CAD were included in this cohort and received standard treatments after admission and followed up.

### Blood sample measurement

Blood samples of all participants were collected after a 10–12 h overnight fasting immediately after their admission. Plasma samples were prepared by centrifugation at 3500 × *g* twice for 10 min each at 15–18 °C and were stored at −80 °C until analysis according to our previous studies [20–22].

Plasma PCSK9 levels were measured using a high-sensitivity, quantitative sandwich enzyme immunoassay (Quantikine ELISA, R&D Systems Europe Ltd) according to our previous study [22]. The lower limit of detection was 0.096 ng/mL. PCSK9 levels of each patient were determined twice and the mean value of the two samples was used in the final analysis. Plasma levels of fibrinogen were quantitatively measured by the method of Clauss and a Stago autoanalyzer with STA Fibrinogen kit (Diagnostic Stago, Taverny, France). The levels of plasma triglyceride (TG), total cholesterol (TC), high-density lipoprotein cholesterol (HDL-C), LDL-C, apolipoprotein (apo) A1, apoB and fasting plasma glucose (FPG) were determined by automatic biochemistry analyser (Hitachi 7150, Japan). Hemoglobin A_1C_ (HbA_1C_) was determined using Tosoh Automated Glycohemoglobin Analyser (HLC-723G8, Tokyo, Japan).

### Follow up

Patients were followed up by telephone and/or clinic revisit every six months, which was conducted by well-trained doctors or nurses. They were blinded to the clinical data of these patients and the purpose of this study until death occurred or the last day of follow-up period. Finally, 63 patients were lost to follow-up, the resulting population consisted of 1225 stable CAD patients taking no lipid-lowering medicines before admission. The following MACEs included hospitalization for unstable angina, coronary revascularization, nonfatal MI, ischemic stroke, and cardiovascular death.

### Diagnosis of clinical disease

DM was considered present if patients met one of the following criteria: self-reported DM, FPG levels ≥ 7.0 mmol/L (126 mg/dL), fasting plasma HbA_1C_ levels ≥ 6.5%, or treated with oral hypoglycemic agents or insulin. Hypertension was defined as systolic blood pressure (BP) ≥ 140 mmHg or diastolic BP ≥ 90 mmHg or the use of anti-hypertensive drugs. Information regarding other diseases, family history and prior therapy of every patient was collected from self-reported medical history. Unstable angina was confirmed if patients appeared rest angina or new-onset severe angina with normal serum levels of cardiac enzymes that required admission. Coronary revascularization included percutaneous coronary intervention (PCI) and coronary artery bypass grafting (CABG) later than 90 days after their discharge. Nonfatal MI was diagnosed as typical angina-like chest pain with increased cardiac troponins or typical electrocardiogram changes. Ischemic stroke was described by acute cerebral infarction symptoms and cerebral diagnostic imaging.

### Calculation of parameters

Body mass index (BMI) was determined as body weight (kg) divided by body height (m^2^). Non-high-density lipoprotein cholesterol (non-HDL-C) was calculated as TC minus HDL-C, triglyceride-rich lipoprotein was calculated as non-HDL-C minus LDL-C as the previous study showed [23]. The value of atherogenic index of plasma was defined as the base 10 logarithm of the ratio of the concentration of TG to HDL-C, the lipoprotein combine index (LCI) was defined as the ratio of TC × TG × LDL-C to HDL-C [24]. Triglyceride glucose was calculated as Ln [fasting TG (mg/dL) × FPG (mg/dL)/2], hemoglobin glycation index (HGI) was calculated as subtracting the predicted HbA_1C_ from the observed HbA_1C_ [25, 26]. The predicted HbA_1C_ was calculated for all participants by inserting the baseline FPG into the subsample linear regression equation derived from another randomly selected 942 patients who admitted to our division [HbA_1C_ = 0.113 × FPG (mg/dL) + 5.289].

### Evaluation of coronary severity

The Gensini score was used to evaluate the severity of coronary stenosis and was calculated as the location score for all diseased segments multiplied by the stenosis score [27], which was performed by two experienced interventional cardiologists according to our previous studies [21, 22].

### Statistical analysis

Continuous variables were expressed as mean ± standard deviation (SD) or median (25th–75th percentile). Categorical variables were expressed as frequencies (percentage). Student’s *t* test or Mann Whitney U-test were used to compare the statistic difference of continuous variables between two groups. χ^2^ analysis and Fisher’s test were performed to determine the statistic difference in categorical variables between the two groups. Spearman correlation analysis was used to evaluate the correlation with plasma PCSK9 levels and CVMMs. The cumulative event-free survival rates of MACEs among subgroups according to PCSK9 levels or/and diabetic status were examined by the Kaplan–Meier curve with the log-rank test. Univariate and multivariate Cox regression analyses were applied to calculate the hazard ratios (HR) of incident MACEs with 95% confidence intervals (CI). A *p* value < 0.05 was considered to be statistically difference. Statistical analysis was performed with Statistical Package for Social Sciences version 25.0.

## Results

### Baseline characteristics

During the follow-up period, 1225 patients firstly diagnosed as stable CAD were enrolled in the current study. The mean age of this population was 57.8 ± 10.1 years, 833 (68%) participants were male, and 377 (30.8%) patients suffered DM. The interquartile PCSK9 levels ranged from 194.79 to 276.13 ng/mL, with a median of 234.52 ng/mL. The baseline characteristics of the cohort were shown in Table 1 according to event occurrence during follow-up. Patients with events had clinical features presented as higher TC, LDL-C, FPG, HbA_1C_ and fibrinogen levels, higher percentage of hypertension, DM and family history of CAD, higher Gensini score and tend to have more multi-diseased coronary vessels (all *p* < 0.05). However, there were no significant differences with regard to the percentage of gender, current smoker and drinker, TG and HDL-C levels, left ventricle ejection fraction and medication prescriptions (except β-blockers) in subjects with and without events. Notably, circulating PCSK9 concentration at baseline was significantly higher in patients with events than that in counterparts [264.26 (219.45, 309.86) *vs* 232.99 (193.72, 274.16) ng/mL, *p* < 0.001]. Meanwhile, we further analyzed the post-admission therapy in all patients and found that there were no significant differences in coronary revascularization including PCI and CABG and medications including lipid-lowering drugs between patients with events and without events (all *p* > 0.05).

**Table 1:** Baseline characteristics in study patients with and without events

		Total (n = 1225)	Non-events (n = 1122)	Events (n = 103)	*p* value
Clinical characteristics					
Age (years)		57.8 ± 10.1	57.7 ± 10.0	58.5 ± 10.8	0.419
Male sex		833 (68.0)	765 (68.3)	68 (66.0)	0.653
BMI (kg/m^2^)		25.9 ± 3.3	25.9 ± 3.2	26.0 ± 4.1	0.704
Hypertension		854 (69.7)	781 (69.6)	73 (70.9)	0.789
Family history of CAD		289 (23.6)	275 (24.5)	14 (13.6)	0.013
Diabetes mellites		377 (30.8)	327 (29.1)	50 (48.5)	< 0.001
Current smoker		481 (39.3)	440 (39.2)	41 (39.8)	0.907
Drinking		284 (23.2)	261 (23.3)	23 (22.3)	0.830
Laboratory parameters					
FPG (mmol/L)		5.9 ± 1.9	5.9 ± 1.8	6.3 ± 3.1	0.042
HbA1C (%)		6.2 ± 1.1	6.2 ± 1.1	6.4 ± 1.1	0.036
ALT (U/L)		20(15,29)	20 (15,29)	20 (14,31)	0.905
Creatinine (μmol/L)		77.7 ± 17.5	77.6 ± 16.9	79.1 ± 23.0	0.408
TC (mmol/L)		4.82 ± 0.96	4.80 ± 0.95	5.02 ± 0.95	0.028
TG (mmol/L)		1.61 (1.16,2.32)	1.6 (1.15,2.31)	1.72 (1.31,2.43)	0.215
HDL-C (mmol/L)		1.09 ± 0.32	1.09 ± 0.32	1.15 ± 0.38	0.060
LDL-C (mmol/L)		3.15 ± 0.85	3.13 ± 0.85	3.35 ± 0.85	0.013
PCSK9 (ng/mL)		234.52 (194.79,276.13)	232.99 (193.72,274.16)	264.26 (219.45,309.86)	< 0.001
Fibrinogen (μg/mL)		3.1 ± 0.8	3.1 ± 0.7	3.3 ± 1.1	0.006
NT-proBNP (pg/mL)		51.9 (31.0,109.3)	50.9 (30.1,111.0)	58.1 (38.1,96.7)	0.172
Diseased vessels					
One vessel		438 (35.8)	411 (36.6)	27 (26.2)	
Two vessels		365 (29.8)	340 (30.3)	25 (24.3)	
Three vessels		422 (34.4)	371 (33.1)	51 (49.5)	
LVEF (%)		64.7 ± 6.7	64.7 ± 6.7	64.8 ± 6.6	0.882
Gensini score		27 (11,44)	26 (10,42)	35.08 (18,60)	0.003
Medications					
Aspirin		464 (37.9)	431 (38.4)	33 (32.0)	0.202
Clopidogrel		130 (10.6)	123 (11.0)	7 (6.8)	0.189
ACEI/ARB		252 (20.6)	235 (20.9)	17 (16.5)	0.286
β-blockers		238 (19.4)	226 (20.1)	12 (11.7)	0.037

*PCSK9* proprotein convertase subtilisin/kexin type 9, *BMI* body mass index, *CAD* coronary artery disease, *FPG* fasting plasma glucose, *HbA*_*1C*_ hemoglobin A_1C_, *ALT* alanine aminotransferase, *TC* total cholesterol, *TG* triglyceride, *HDL-C* high-density lipoprotein cholesterol, *LDL-C* low-density lipoprotein cholesterol, *NT-proBNP* N-Terminal pro-brain natriuretic peptide, *LVEF* left ventricular ejection fraction, *ACEI* angiotensin converting enzyme inhibitors, *ARB* angiotensin receptor blockers, *p* < 0.05 suggests significant difference

Next, we divided the entire study population into two groups according to the median of PCSK9 levels at baseline (shown in Table 2). Patients in low PCSK9 group were younger, female, and more likely to be current smoker and drinker (all *p* < 0.05). Furthermore, baseline levels of plasma TC, LDL-C, HDL-C, FPG, HbA_1C_ and fibrinogen were significantly higher in participants with high PCSK9 levels compared to those with low PCSK9 levels (all *p* < 0.05). Besides, BMI, percentage of hypertension and DM, TG levels, Gensini score and medication use were no significant differences between two groups (all *p* < 0.05). Additionally, as presented in Additional file 1: Table S1, we also found that CAD subjects with DM had higher PCSK9, TG levels and Gensini score, lower HDL-C levels, and more likely to occur multi-diseased coronary vessels compared with nondiabetic patients (all *p* < 0.05).

**Table 2:** Baseline characteristics of patients according to PCSK9 stratification

Variables	Total (n = 1225)	PCSK9 concentration (ng/mL)		*p* value
		< 234.52 (n = 612)	≥ 234.52 (n = 613)	
Clinical characteristics				
Age (years)	57.8 ± 10.1	57.1 ± 10.1	58.4 ± 10.0	0.027
Male sex	833 (68.0)	469 (76.6)	364 (59.4)	< 0.001
BMI (kg/m^2^)	25.9 ± 3.3	26.0 ± 3.5	25.8 ± 3.2	0.217
Hypertension	854 (69.7)	424 (69.3)	430 (70.1)	0.742
Family history of CAD	289 (23.6)	126 (20.6)	163 (26.6)	0.013
Diabetes mellites	377 (30.8)	180 (29.4)	197 (32.1)	0.301
Current smoker	481 (39.3)	258 (53.6)	223 (46.4)	0.038
Drinking	284 (23.2)	159 (56.0)	125 (44.0)	0.020
Laboratory parameters				
FPG (mmol/L)	5.9 ± 1.9	5.7 ± 1.7	6.0 ± 2.1	0.012
HbA_1C_ (%)	6.2 ± 1.1	6.1 ± 1.0	6.3 ± 1.2	0.010
ALT(U/L)	20 (15,29)	20 (15,28)	20 (14,30)	0.769
Creatinine (μmol/L)	77.7 ± 17.5	77.9 ± 16.1	77.5 ± 18.8	0.680
TC (mmol/L)	4.82 ± 0.96	4.67 ± 0.90	4.98 ± 0.99	< 0.001
TG (mmol/L)	1.61 (1.16,2.31)	1.55 (1.12,2.30)	1.65 (1.20,2.34)	0.226
HDL-C (mmol/L)	1.09 ± 0.32	1.07 ± 0.33	1.12 ± 0.32	0.011
LDL-C (mmol/L)	3.15 ± 0.85	3.00 ± 0.78	3.29 ± 0.89	< 0.001
Fibrinogen (μg/mL)	3.1 ± 0.8	3.0 ± 0.6	3.2 ± 0.8	< 0.001
NT-proBNP (pg/mL)	51.9 (31.0,109.3)	49.45 (31.2,92.5)	53.2 (30.4,130.0)	0.083
Diseased vessels				
One vessel	438 (35.8)	241 (39.4)	197 (32.1)	
Two vessels	365 (29.8)	173 (28.2)	192 (31.3)	
Three vessels	422 (34.4)	198 (32.4)	224 (36.5)	
LVEF (%)	64.7 ± 6.7	64.6 ± 6.9	64.8 ± 6.6	0.716
Gensini score	27 (11,44)	25 (10,43)	28 (12,44)	0.095
Medications				
Aspirin	464 (37.9)	228 (37.3)	236 (38.5)	0.654
Clopidogrel	130 (10.6)	61 (10.0)	69 (11.3)	0.464
ACEI/ARB	252 (20.6)	119 (19.4)	133 (21.7)	0.330
β-blockers	238 (19.4)	108 (17.6)	130 (21.2)	0.115
*MACEs*	103 (8.4)	36 (5.9)	67 (10.9)	0.001

*PCSK9* proprotein convertase subtilisin/kexin type 9, *BMI* body mass index, *CAD* coronary artery disease, *FPG* fasting plasma glucose, *HbA*_*1C*_ hemoglobin A_1C_, *ALT* alanine aminotransferase, *TC* total cholesterol, *TG* triglyceride, *HDL-C* high-density lipoprotein cholesterol, *LDL-C* low-density lipoprotein cholesterol, *NT-proBNP* N-Terminal pro-brain natriuretic peptide, *LVEF* left ventricular ejection fraction, *ACEI* angiotensin converting enzyme inhibitors, *ARB* angiotensin receptor blockers, *MACEs* major adverse cardiovascular events, *p* < 0.05 suggests significant difference

### Association of PCSK9 with CVMMs

Spearman correlation analysis showed that plasma PCSK9 levels were significantly and positively associated with multiple CVMMs, especially with glycemic and lipid parameters including traditional, novel and derived lipid indicators (shown in Table 3) in CAD patients. Furthermore, our results found a significantly positive correlation of PCSK9 with novel derived CVMMs including LCI and HGI in stable CAD patients (r = 0.112, *p* < 0.0001; r = 0.112, *p* < 0.0001, respectively). Meanwhile, the further analysis suggested that positive relationships between PCSK9 and several lipid-related markers including TC, LDL-C, non-HDL-C, apoA1 and apoB remained in DM group (r = 0.133, *p* = 0.010; r = 0.153, *p* = 0.003; r = 0.104, *p* = 0.043; r = 0.104, *p* = 0.044; r = 0.138, *p* = 0.007, respectively) and non-DM group (r = 0.234, *p* < 0.0001; r = 0.215, *p* < 0.0001; r = 0.217, *p* < 0.0001; r = 0.090, *p* = 0.009; r = 0.213, *p* < 0.0001, respectively). Additionally, PCSK9 levels were positively associated with HbA_1C_ in DM patients (r = 0.111, *p* = 0.032), which was consistent with our previous study [18]. A weak correlation of PCSK9 and HbA_1C_ was also observed in non-DM group (r = 0.098, *p* = 0.004).

**Table 3:** Spearman correlation analysis between PCSK9 and cardiovascular metabolic makers

Variables	Overall		DM		Non-DM	
	n = 1225		n = 377		n = 848	
	r	*p*	r	*p*	r	*p*
Traditional lipid parameters						
TC	0.199	< 0.0001	0.133	0.010	0.234	< 0.0001
TG	0.063	0.028	0.010	0.851	0.078	0.023
HDL-C	0.096	0.001	0.098	0.057	0.100	0.003
LDL-C	0.195	< 0.0001	0.153	0.003	0.215	< 0.0001
Novel lipid markers						
non-HDL-C	0.180	< 0.0001	0.104	0.043	0.217	< 0.0001
Lp(a)	0.062	< 0.0001	0.079	0.126	0.054	0.117
ApoA1	0.092	0.001	0.104	0.044	0.090	0.009
ApoB	0.192	< 0.0001	0.138	0.007	0.213	< 0.0001
TRLP	0.045	0.116	0.046	0.371	0.038	0.264
Derived lipid indicators						
TG/HDL-C	0.012	0.682	−0.032	0.538	0.023	0.510
LDL-C/HDL-C	0.073	0.010	0.026	0.620	0.089	0.009
TC/HDL-C	0.037	0.195	−0.019	0.712	0.056	0.102
ApoB/ApoA1	0.075	0.009	0.034	0.510	0.088	0.011
non-HDL-C/TC	0.037	0.195	−0.019	0.712	0.056	0.102
AIP	0.012	0.682	−0.032	0.538	0.023	0.510
LCI	0.112	< 0.0001	0.042	0.414	0.139	< 0.0001
Other metabolic indexes						
SBP	−0.014	0.632	−0.001	0.988	−0.033	0.343
DBP	−0.039	0.169	−0.059	0.252	−0.035	0.315
BMI	−0.007	0.815	−0.042	0.417	−0.008	0.818
Glucose	0.104	< 0.0001	0.088	0.087	0.087	0.012
HbA_1C_	0.123	< 0.0001	0.111	0.032	0.098	0.004
HGI	0.112	< 0.0001	0.076	0.141	0.085	0.013
TyG	0.088	0.002	0.040	0.435	0.087	0.011

*PCSK9* proprotein convertase subtilisin/kexin type 9, *CAD* coronary artery disease, *DM* diabetes mellitus, *non-DM* non-diabetes mellitus, *TC* total cholesterol, *TG* triglyceride, *HDL-C* high-density lipoprotein cholesterol, *LDL-C* low-density lipoprotein cholesterol, *non-HDL-C* non-high-density lipoprotein cholesterol, *ApoA1* Apolipoprotein A1, *ApoB* apolipoprotein B, *TRLP* triglyceride-rich lipoproteins, *AIP* atherogenic index of plasma, *LCI* lipoprotein combine index, *SBP* systolic blood pressure, *DBP* diastolic blood pressure, *BMI* body mass index, *HbA*_*1C*_ hemoglobin A_1C_, *HGI* hemoglobin glycation index, *TyG* triglyceride glucose, *p* < 0.05 suggests significant difference. (TRLP = TC—HDL-C—LDL-C; LCI = TC ∗ TG ∗ LDL/HDL-C; AIP = log (TG/HDL-C); TyG = Ln (TG*FPG/2); HGI = observed HbA1c—predicted HbA1c), *p* < 0.05 suggests significant difference

### PCSK9, DM and cardiovascular outcomes

**Fig. 2:**
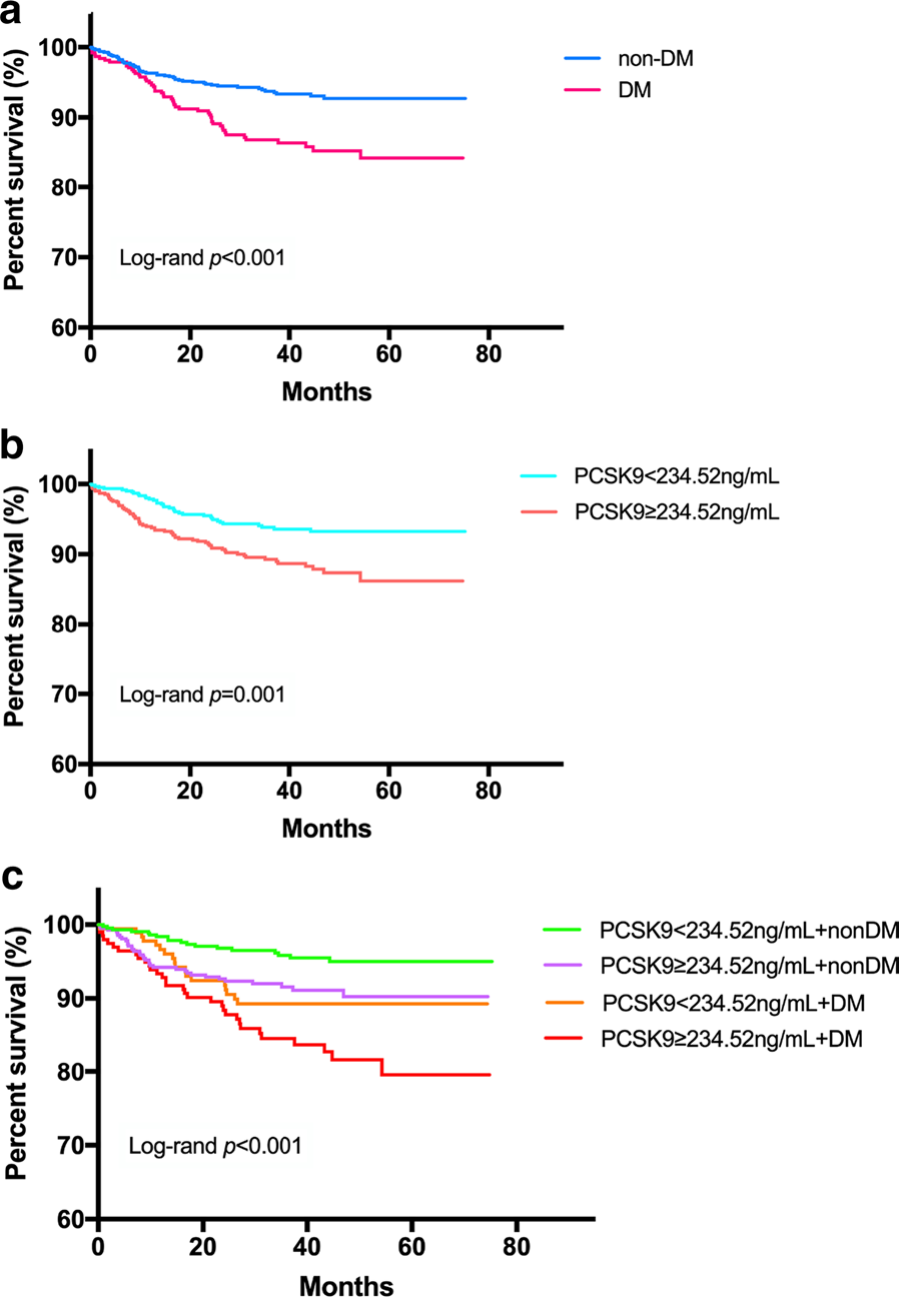
Kaplan–Meier analysis according to different diabetic status (**a**), different PCSK9 levels (**b**), and status of both PCSK9 levels and diabetic status (**c**). *PCSK9* proprotein convertase subtilisin/kexin type 9, *non-DM* non-diabetes mellitus, *DM* diabetes mellitus

Over a median of 3.3 years’ follow-up, 103 patients developed subsequent MACEs (3 suffered nonfatal MI, 41 underwent coronary revascularization, 8 had ischemic stroke, 6 died and 45 experienced hospitalization because of unstable angina pectoris). High PCSK9 group had higher incidence of evens during follow-up time compared to low PCSK9 group [(67 (10.9%) *vs* 36 (5.9%), *p* = 0.001)], while data from Additional file 1: Table S1, showed that DM group had higher prevalence of MACEs than non-DM group [50 (13.3%) *vs* 50 (6.3%), *p* < 0.001]. Kaplan Meier curve with the log-rank test displayed that patients in high PCSK9 group had lower event-free survival rate compared with those in low PCSK9 group (*p* = 0.001, Fig. 2b) and DM subjects were more likely to occur future worse cardiovascular events compared to patients without DM (*p* < 0.001, Fig. 2a). Hereafter, participants were categorized into 4 subgroups when combined diabetic status with PCSK9 levels, those with high PCSK9 levels had significantly higher risk of MACEs than that with reference subgroup (non-DM plus low PCSK9 levels) despite of diabetic or nondiabetic population. DM plus high PCSK9 levels subgroup had the lowest cumulative event-free survival rate among the four subgroups (*p* < 0.001, Fig. 2c).

**Fig. 3:**
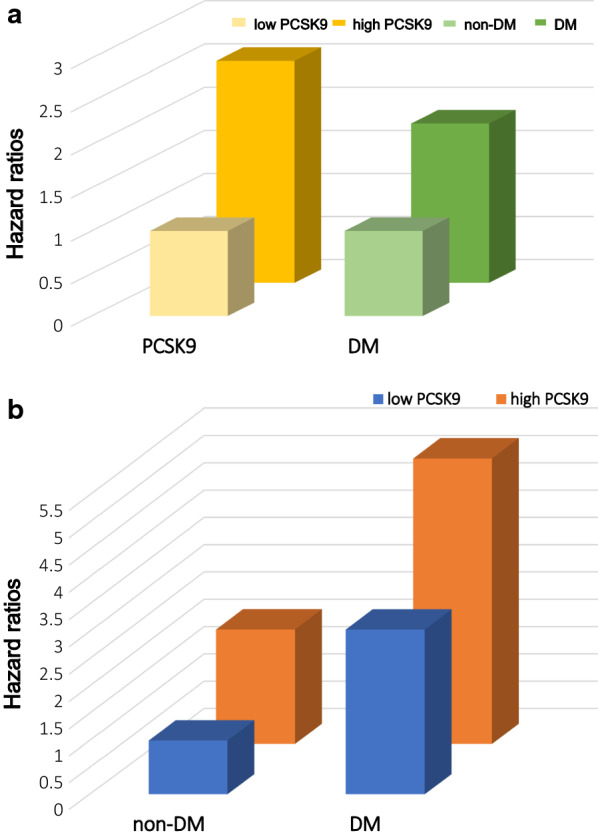
Risk of major adverse cardiovascular events to different diabetic status or PCSK9 levels (**a**), and the combination of diabetic status and PCSK9 levels (**b**). Adjusting for age, sex, body mass index, smoking, drinking, hypertension, family history of coronary artery disease, Gensini score, total cholesterol, low density lipoprotein cholesterol, high density lipoprotein cholesterol, triglyceride, fasting plasma glucose, hemoglobin A_1c_, fibrinogen and β-blockers

As presented in the Additional file 1: Table S2, univariate Cox regression analysis indicated that DM patients had 2.13-fold higher risk of MACEs than those without DM (HR: 2.130, 95% CI: 1.447–3.135, *p* < 0.01). After adjusting for confounding factors, the significant association still remained (Additional file 1: Table S2 and Fig. 3a). Meanwhile, the high PCSK9 group had significantly higher risk of MACEs than those in low PCSK9 group in this entire population (adjusted HR: 1.855, 95% CI: 1.215–2.831, *p* < 0.01, Additional file 1: Table S2 and Fig. 3a). Notably, as a continuous variable, per 1-SD increase of PCSK9 level was significantly associated with 36.1 and 45.8% increased risk for MACEs in DM and non-DM groups, respectively (adjusted HR: 1.361, 95% CI: 1.037–1.785, *p* < 0.01; adjusted HR: 1.458, 95% CI: 1.128–1.885, *p* < 0.05, respectively, Table 4 and Fig. 3b). Likewise, in the diabetic group, there was 2.294-fold (95% CI: 1.230–4.278) higher risks of MACEs in patients with high PCSK than those with low PCSK9 after adjustment for established cardiovascular metabolic factors. Interestingly, multivariate Cox regression analysis based on the combination of diabetic status and PCSK9 levels revealed that patients in DM plus high PCSK9 levels, DM plus low PCSK9 levels and non-DM plus high PCSK9 levels groups had 5.233-fold (95% CI: 2.546–10.757), 3.033-fold (95% CI: 1.442–6.376) and 2.1-fold (95% CI: 1.174–3.757) higher risk of MACEs (all *p* < 0.05, Table 4 and Fig. 3b) than reference subgroup, respectively.

**Table 4:** PCSK9 levels in relation to MACEs in patients with different diabetic status

PCSK9 (ng/mL)	HR (95% CI)		
	Crude model	Model 1	Model 2
Non-DM			
PCSK9 per-SD increase	1.543 (1.224–1.945)^b^	1.571 (1.232–2.004)^b^	1.458 (1.128–1.885)^b^
PCSK9 < 234.52 ng/mL	1.0 (Reference)	1.0 (Reference)	1.0 (Reference)
PCSK9 ≥ 234.52 ng/mL	2.137 (1.210–3.775)^b^	2.140 (1.206–3.795)^b^	2.100 (1.174–3.757)^a^
DM			
PCSK9 per-SD increase	1.258 (0.975–1.623)	1.262 (0.972–1.639)	1.361 (1.037–1.785)^a^
PCSK9 < 234.52 ng/mL	2.430 (1.264–4.467)^b^	2.424 (1.260–4.663)^b^	3.033 (1.442–6.376)^b^
PCSK9 ≥ 234.52 ng/mL	4.092 (2.297–7.290)^b^	4.084 (2.274–7.333)^b^	5.233 (2.546–10.757)^b^

*MACEs* major adverse cardiovascular events, *non-DM* diabetes mellitus, *non-DM* non-diabetes mellitus, *CI* confidence interval, *HR* hazard ratio, Model 1 adjusted for age and sex; Model 2 adjusted for model 1, body mass index, smoking, drinking, hypertension, family history of coronary artery disease, Gensini score, total cholesterol, low density lipoprotein cholesterol, high density lipoprotein cholesterol, triglyceride, fasting plasma glucose, hemoglobin A_1c_, fibrinogen and β-blockers

^a^for *p* < 0.05

^b^for *p* < 0.01

## Discussion

In this study, we investigated the association of PCSK9 with CVMMs and the predictive value of PCSK9 for cardiovascular events in stable CAD patients with DM or without DM. Firstly, we found that plasma PCSK9 levels were significantly correlated with LCI and HGI, the novel CVMMs related to cardiovascular risks, in all CAD patients and positively associated with lipid- and glucose-related CVMMs including TC, LDL-C, non-HDL-C and HbA_1C_ in DM participants with stable CAD. Besides, our results showed that circulating PCSK9 concentration at baseline was positively associated with the risk of MACEs in diabetic patients with stable CAD even after adjusting for established cardiometabolic factors. Finally, data also indicated that the combination of high PCSK9 levels and DM had significantly higher cardiovascular events in our studied patients. To the best of our knowledge, this is the first study showing that PCSK9 can predict worse clinical cardiovascular outcomes in diabetic patients with stable CAD and the combination of plasma PCSK9 levels and diabetes status enhanced predictive value in CAD patients.

DM has become the one of major worldwide healthy issues and its prevalence has been rapidly growing in the past decades [28]. Of note, DM patients are more likely to have higher risk of cardiovascular events when accompanied by other metabolic disorders, especially in patients with ASCVD [1, 4]. Furthermore, atherogenic dyslipidemia has been reported to have an essential role in the prognosis of these two diseases, DM and ASCVD [29]. Even if receiving standard treatment, diabetic patients with CAD still have high cardiovascular risk. Therefore, further knowledge of cardiovascular risks related to diabetic patients with CAD will be helpful for better prevention and management for such kind of patients.

The best-known function of PCSK9 is posttranslational regulation of LDLR on hepatocytes to clear LDL-C from the circulation [30]. In recent years, it has been reported that PCSK9 is linked to atherosclerosis and become an attractive treatment target for CAD [10, 15, 27]. Meanwhile, PCSK9 levels were associated with several CVMMs which have been proposed as predictor of cardiovascular risks [18, 24–26]. However, the predictive role of PCSK9 on cardiovascular outcomes is currently controversial. A positive association with PCSK9 and cardiovascular events was found in the general population, in FH subjects, and in patients with acute coronary syndrome or stable CAD [15, 16, 20, 31]. In contrast, a prospective cohort study of primary prevention included 716 initially healthy American women and displayed that the baseline plasma levels of PCSK9 cannot predict future cardiovascular events [32]. Moreover, the SURDIAGENE study found a similar result in DM population [33]. Hence, whether PCSK9 can be a novel predictor of prognosis and its correlation with metabolic factors need more investigations, especially in CAD patients with or without DM.

The associations of PCSK9 with cardiovascular metabolic parameters including lipid- and diabetes-related indicators, BMI and BP, have been found in different populations [17–19]. In patients with stable CAD, low plasma levels of PCSK9 were linked with a particular metabolic phenotype (low HDL-C, the metabolic syndrome, obesity, insulin resistance and DM) [19]. In CAD patients with DM who had high non-HDL-C/LDL-C levels, PCSK9 inhibitor significantly reduced atherogenic cholesterol and LDL-particle number versus control [34]. What’s more, an animal study using obese-insulin resistant rat model, found that PCSK9 inhibition attenuated metabolic impairment and also ameliorated cardiovascular dysfunction more efficiently than atorvastatin [35]. In the current study, our results also showed a significant correlation between plasma PCSK9 levels and multiple CVMMs including traditional, novel and derived parameters in CAD patients with DM or without DM. Interestingly, in whole stable CAD patients, we found that PCSK9 was significantly and positively related to LCI and HGI, two novel lipid- or glycemic derived indicators and the potential cardiovascular outcomes predictors.[26, 36]. It was worth mentioning that, we did not find the association between PCSK9 and some indictors, mainly HDL-C, TG and their derived indexes in DM patients, which may be due to the higher heterogeneous in metabolic phenotypes. In addition, the present study showed that patients with high plasma PCSK9 concentration were more likely to be female, which was consistent with previous studies [37, 38]. The reason for this phenomenon may be that plasma PCSK9 levels are influenced by hormones such as estrogen [39]. Besides, we found that low PCSK9 levels were associated with the feature of smoking and drinking was inconsistent with other studies [40, 41], which may need to investigate further in a large sample study.

In literature, the close relation between PCSK9 and DM has been documented. The results of available epidemiology and clinical studies showed a higher PCSK9 levels in diabetic patients [17, 18]. Moreover, it is well-established that insulin resistance is the main cause of DM. Previous data suggested that insulin resistance and subsequent hyperinsulinemia could enhance PCSK9 expression and increased plasma levels of PCSK9 were related to poor glycemic control in DM [42]. Besides, it has been demonstrated that insulin resistance plays a pivotal role in PCSK9 homeostasis in severely obese patients [43]. PCSK9 critically controls LDLR expression in pancreas perhaps contributing to the maintenance of a proper physiological balance to limit cholesterol overload in beta cells [44]. Regarding pharmacology, it has been found that liraglutide can suppress PCSK9 expression through hepatocyte nuclear factor 1 alpha-dependent mechanism in HepG2 cells and db/db mice [45]. In addition, as found above in this study, PCSK9 was significantly correlated with glycemic parameters including FPG and HbA_1C_. Meanwhile, our results indicated that PCSK9 or DM was independently predictors for MACEs respectively. The PCSK9 levels categorized according to its median and as a continuous variable were all independently related to poor outcomes even after adjustment for established cardiovascular risk factors in DM patients with CAD. Clearly, we not only analyzed the prognostic ability of PCSK9 but also further demonstrated the combined impact of elevated PCSK9 levels plus diabetic status. When added PCSK9 stratification and diabetic status to stratifying factors, patients in high PCSK9 group appeared to have the highest risk of subsequent cardiovascular events with DM (HR: 5.233, 95% CI: 2.546–10.757). These findings suggested that the measurement of PCSK9 might be useful to predict the occurrence of MACEs in diabetic patients with CAD and high PCSK9 levels plus DM could enhance the prediction of worse clinical outcomes.

The previous studies including ours have demonstrated that some lipid-lowering medications, such as statins, ezetimibe, can up-regulated the expression of PCSK9 genes and increased the circulating concentration of PCSK9 [46–48]. A meta-analysis of 15 clinical trials have demonstrated a significant increase in plasma PCSK9 levels after statin therapy, irrespective of the type of statin [49].Besides, recent data have indicated that PCSK9 inhibitors can significantly reduce circulating PCSK9 levels [48]. That is the reason why we chose the diabetic patients with stable CAD, who did not receive any lipid-lowering therapy before admission as the participants to performed such study [48, 50].

Nevertheless, several limitations must be given consideration. First of all, the sample size and follow-up time of the current study were relatively small and short, which could lead to insufficient statistical power in analyses of subgroups. Moreover, due to fewer events, we could not further perform subgroup analyses for nonfatal MI, stroke and cardiovascular death. Secondly, we only measured the baseline of plasma PCSK9 levels and did not evaluate the effects of its longitudinal change (especially the effects of lipid-lowering drugs on PCSK9 levels after discharge) on cardiovascular events during follow-up time. Lastly, as with the feature of any observational and prospective study, only association but no causal link could be determined. Further prospective studies with larger sample size and long-term follow-up are required to confirm our findings.

## Conclusion

In conclusion, our data, for the first time, showed that PCSK9 was associated with multiple CVMMs (including TC, LDL-C, non-HDL-C and HbA_1C_). Elevated PCSK9 levels were an independent predictor for adverse cardiovascular outcomes in diabetic patients with stable CAD and the combination of high PCSK9 and DM could increase predicting ability for cardiovascular events.

## Supplementary information

**Additional file 1: Table S1.** Baseline characteristics of patients with DM and non-DM. **Table S2.** Cox regression analysis of MACEs according to different diabetic status and PCSK9 levels. **Table S3.** Therapy after admission of patients with events and without events.

## Data Availability

The datasets used and analyzed during the current study are available from the corresponding author on reasonable request.

## Abbreviations

DM: Diabetes mellitus
CAD: Coronary artery disease
ASCVD: Atherosclerotic cardiovascular disease
LDL-C: Low-density lipoprotein cholesterol
PCSK9: Proprotein convertase subtilisin/kexin type 9
LDLR: Low-density lipoprotein cholesterol receptors
CVMMs: Cardiovascular metabolic markers
MACEs: Major adverse cardiovascular events
MI: Myocardial infarction
FH: Familial hypercholesterolemia
TG: Triglyceride
TC: Total cholesterol
HDL-C: High-density lipoprotein cholesterol
apo: Apolipoprotein
FPG: Fasting plasma glucose
HbA_1C_: Hemoglobin A_1C_
BP: Blood pressure
PCI: Percutaneous coronary intervention
CABG: Coronary artery bypass grafting
BMI: Body mass index
non-HDL-C: Non-high-density lipoprotein cholesterol
LCI: Lipoprotein combine index
HGI: Hemoglobin glycation index
SD: Standard deviation
HR: Hazard ratios
CI: Confidence intervals
