# Supplementary material for: Association of circulating PCSK9 concentration with cardiovascular metabolic markers and outcomes in stable coronary artery disease patients with or without diabetes: a prospective, observational cohort study
Source: Cardiovasc Diabetol. 2020 Oct 6;19:167. doi: 10.1186/s12933-020-01142-0 (PMC7541318; doi:10.1186/s12933-020-01142-0)
Supplement: Supplementary file 1 — Additional file 1: Table S1. Baseline characteristics of patients with DM and non-DM. Table S2. Cox regression analysis of MACEs according to different diabetic status and PCSK9 levels. Table S3. Therapy after admission of patients with events and without events. [file 12933_2020_1142_MOESM1_ESM.docx]

**Table S1. Baseline characteristics of patients with DM and non-DM.**

|  | | **Total** | |  | |  | | **non-DM** | |  | | **DM** |  | |  | |
| --- | --- | --- | --- | --- | --- | --- | --- | --- | --- | --- | --- | --- | --- | --- | --- | --- |
| **Variables** | | **(n=1225)** |  | | | |  | | **(n=848)** |  | | **(n=377)** |  | ***P* value** | | |
| **Clinical characteristics** | | | | |  | | | | | |  | |  | | |  |
| Age (years) | 57.8±10.1 | | | | 57.1±10.4 | | | | | | 59.2±9.3 | | 0.001 | | |  |
| Male sex | 833(68.0) | | | | 579(68.3) | | | | | | 254(67.4) | | 0.754 | | |  |
| BMI (kg/m^2^) | 25.9±3.3 | | | | 25.6±3.2 | | | | | | 26.6±3.4 | | <0.001 | | |  |
| Hypertension | 854(69.7) | | | | 559(65.9) | | | | | | 295(78.2) | | <0.001 | | |  |
| Family history of CAD | 289(23.6) | | | | 206(24.3) | | | | | | 83(22.0) | | 0.386 | | |  |
| Current smoker | 481(39.3) | | | | 339(40.0) | | | | | | 142(37.7) | | 0.445 | | |  |
| Drinking | 284(23.2) | | | | 209(24.6) | | | | | | 75(19.9) | | 0.069 | | |  |
| **Laboratory parameters** | | | | |  | | | | | |  | |  | | |  |
| FPG (mmol/L) | 5.9±1.9 | | | | 5.1±0.6 | | | | | | 7.6±2.7 | | <0.001 | | |  |
| HbA1C (%) | 6.2±1.1 | | | | 5.7±0.4 | | | | | | 7.4±1.3 | | <0.001 | | |  |
| ALT(U/L) | 20(15,29) | | | | 20(15,28) | | | | | | 21(15,31) | | 0.394 | | |  |
| Creatinine (μmol/L) | 77.7±17.5 | | | | 77.5±16.8 | | | | | | 78.1±19.0 | | 0.628 | | |  |
| TC (mmol/L) | 4.82±0.96 | | | | 4.82±0.92 | | | | | | 4.83±1.03 | | 0.834 | | |  |
| TG (mmol/L) | 1.61(1.16,2.32) | | | | 1.57(1.12,2.24) | | | | | | 1.68(1.28,2.4) | | 0.004 | | |  |
| HDL-C (mmol/L) | 1.09±0.32 | | | | 1.11±0.33 | | | | | | 1.06±0.30 | | 0.016 | | |  |
| LDL-C (mmol/L) | 3.15±0.85 | | | | 3.15±0.84 | | | | | | 3.14±0.88 | | 0.847 | | |  |
| PCSK9 (ng/mL) | 234.52(194.79,276.13) | | | | 233.48(193.25,271.24) | | | | | | 246.51(199.43,292.22) | | 0.005 | | |  |
| Fibrinogen (μg/mL) | 3.1±0.8 | | | | 3.1±0.8 | | | | | | 3.3±0.7 | | <0.001 | | |  |
| NT-proBNP (pg/mL) | 51.9(31.0,109.3) | | | | 49.5(29.5,105.1) | | | | | | 54.5(35.1,118.1) | | 0.043 | | |  |
| **Diseased vessels** | | | | |  | | | | | |  | |  | | |  |
| One vessel | 438(35.8) | | | | 328(38.7) | | | | | | 110(29.2) | |  | | |  |
| Two vessels | 365(29.8) | | | | 261(30.8) | | | | | | 104(27.6) | |  | | |  |
| Three vessels | 422(34.4) | | | | 259(30.5) | | | | | | 163(43.2) | |  | | |  |
| LVEF (%) | 64.7±6.7 | | | | 65.0±6.8 | | | | | | 64.0±6.6 | | 0.023 | | |  |
| Gensini score | 27(11,44) | | | | 24(10,40) | | | | | | 34(16,57) | | <0.001 | | |  |
| **Medications** | | | | |  | | | | | |  | |  | | |  |
| Aspirin | 464(37.9) | | | | 310(36.6) | | | | | | 154(40.8) | | 0.153 | | |  |
| Clopidogrel | 130(10.6) | | | | 85(10.0) | | | | | | 45(11.9) | | 0.316 | | |  |
| ACEI/ARB | 252(20.6) | | | | 156(18.4) | | | | | | 96(25.5) | | 0.005 | | |  |
| β-blockers | 238(19.4) | | | | 157(18.5) | | | | | | 81(21.5) | | 0.225 | | |  |
| **MACEs** | 103(8.4) | | | | 53(6.3) | | | | | | 50(13.3) | | <0.001 | | |  |

PCSK9, proprotein convertase subtilisin/kexin type 9; DM, diabetes mellitus; non-DM, non-diabetes mellitus; BMI, body mass index; CAD, coronary artery disease; FPG, fasting plasma glucose; HbA_1C_, hemoglobin A_1C_; ALT, alanine aminotransferase; TC, total cholesterol; TG, triglyceride; HDL-C, high-density lipoprotein cholesterol; LDL-C, low-density lipoprotein cholesterol; NT-proBNP, N-Terminal pro-brain natriuretic peptide; LVEF, left ventricular ejection fraction; ACEI, angiotensin converting enzyme inhibitors; ARB, angiotensin receptor blockers; MACEs, major adverse cardiovascular events, *p*<0.05 suggests significant difference.

**Table S2. Cox regression analysis of MACEs according to different diabetic status and PCSK9 levels.**

|  |  | | **HR (95%CI)** | |  |
| --- | --- | --- | --- | --- | --- |
|  | **Crude model** | **Model 1** | | **Model 2** | |
| **Diabetic status** |  |  | |  | |
| non-DM | 1.0(Reference) | 1.0(Reference) | | 1.0(Reference) | |
| DM | 2.130(1.447-3.135)^b^ | 2.117(1.435-3.123)^b^ | | 2.578(1.504-4.418)^b^ | |
| **PCSK9 levels** |  |  | |  | |
| PCSK9 per-SD increase | 1.430(1.210-1.691)^b^ | 1.443(1.214-1.716)^b^ | | 1.370(1.148-1.635)^b^ | |
| PCSK9 < 234.52ng/mL | 1.0(Reference) | 1.0(Reference) | | 1.0(Reference) | |
| PCSK9≥234.52ng/mL | 1.955(1.303-2.933)^b^ | 1.950(1.291-2.945)^b^ | | 1.855(1.215-2.831)^b^ | |

MACEs: major adverse cardiovascular events; non-DM: non-diabetes mellitus; DM: diabetes mellitus; CI: confidence intervals; HR: hazard ratios; Model 1 adjusted for age and sex; model 2 adjusted for age, sex, body mass index, smoking, drinking, hypertension, family history of coronary artery disease, Gensini score, total cholesterol, low density lipoprotein cholesterol, high density lipoprotein cholesterol, triglyceride, fasting plasma glucose, hemoglobin A_1c_, fibrinogen and β-blockers, ^a^ for *p*<0.05, ^b^ for *p*<0.01.

**Table S3. Therapy after admission of patients with events and without events.**

|  | **Total** | **non-events** | **Events** |  |
| --- | --- | --- | --- | --- |
|  | **(n=1225)** | **(n=1122)** | **(n=103)** | ***p* value** |
| Revascularization | 517(42.2) | 476(42.4) | 41(39.8) | 0.607 |
| Statin | 1141(93.1) | 1047(93.3) | 94(91.3) | 0.430 |
| Aspirin | 930(75.9) | 846(75.4) | 84(81.6) | 0.162 |
| Clopidogrel | 741(60.5) | 671(59.8) | 70(68.0) | 0.105 |
| ACEI/ARB | 390(31.8) | 351(31.3) | 39(37.9) | 0.170 |
| β-blockers | 691(56.4) | 627(55.9) | 64(62.1) | 0.221 |

Revascularization included percutaneous coronary intervention and coronary artery bypass graft after admission. ACEI, angiotensin converting enzyme inhibitors; ARB, angiotensin receptor blockers, *p* < 0.05 suggests significant difference.
